# Limit case analysis of the “stable indenter velocity” method for obtaining creep stress exponents from constant load indentation creep tests

**DOI:** 10.1007/s11043-016-9316-x

**Published:** 2016-06-21

**Authors:** J. Campbell, J. Dean, T. W. Clyne

**Affiliations:** 1 Department of Materials Science & Metallurgy, Cambridge University, 27 Charles Babbage Road, Cambridge, CB3 0FS UK; 2 Double Precision Consultancy, Salisbury House, Station Road, Cambridge, CB1 2LA, UK

**Keywords:** Instrumented indentation, Creep

## Abstract

This study concerns a commonly-used procedure for evaluating the steady state creep stress exponent, $n$, from indentation data. The procedure involves monitoring the indenter displacement history under constant load and making the assumption that, once its velocity has stabilised, the system is in a quasi-steady state, with stage II creep dominating the behaviour. The stress and strain fields under the indenter are represented by “equivalent stress” and “equivalent strain rate” values. The estimate of $n$ is then obtained as the gradient of a plot of the logarithm of the equivalent strain rate against the logarithm of the equivalent stress. Concerns have, however, been expressed about the reliability of this procedure, and indeed it has already been shown to be fundamentally flawed. In the present paper, it is demonstrated, using a very simple analysis, that, for a genuinely stable velocity, the procedure always leads to the same, constant value for $n$ (either 1.0 or 0.5, depending on whether the tip shape is spherical or self-similar). This occurs irrespective of the value of the measured velocity, or indeed of any creep characteristic of the material. It is now clear that previously-measured values of $n$, obtained using this procedure, have varied in a more or less random fashion, depending on the functional form chosen to represent the displacement–time history and the experimental variables (tip shape and size, penetration depth, etc.), with little or no sensitivity to the true value of $n$.

## Introduction

It has been shown (Guelorget et al. 2007; Lee et al. 2008; Heinrich et al. 2009; Dean et al. 2010, 2013) that inverse FEM modelling procedures can be used to infer material property values, including plasticity and creep characteristics, from instrumented indentation data, although it should be recognised that considerable work remains to be done (on identification of optimal comparator data sets, implementation of convergence criteria and development of user-friendly software packages) before the approach can be widely regarded as reliable and convenient. Nevertheless, the procedures, which in the case of creep can take full account of the roles of primary and secondary regimes of behaviour, are basically sound and, in view of the considerable attractions of indentation testing, it seems likely that this approach will ultimately lead to the wide availability and utilisation of tractable tools and procedures for these purposes.

In the meantime, there has been a strong motivation to develop analytical methods of interpreting indentation data so as to obtain material property values. Such methodology is well-established for the Young’s modulus and reliable expressions are also available for the yield stress, at least when the effect of work hardening can be neglected. Moreover, a simple analytical procedure has evolved for derivation of the steady-state creep stress exponent ($n$) from indentation creep dwell data. The analysis is based on identifying “equivalent” values for both the stress and the strain rate beneath the indenter. The equivalent stress is taken to be the load over the projected area, while the equivalent strain rate is taken as the indenter velocity $(\mathrm{d} h/\mathrm{d}t)$ over the current indenter depth ($h$). It is assumed that steady-state (stage II) creep is rapidly established throughout the deforming volume, and that this condition applies as soon as the indenter velocity has become constant. Closely-related procedures are sometimes employed that are based on a “strain rate sensitivity” formulation.

It is perhaps worth mentioning that, while the common assumption has been that a “steady state” is attained when the indenter velocity has become constant, one early treatment (Lucas and Oliver 1999) was based on the concept of actually fixing the “indentation strain rate” (i.e. $(\mathrm{d}h/\mathrm{d}t)/h$). It would be very unlikely for this condition to be satisfied under “normal” conditions (i.e. with a fixed applied load), since it would require the penetration velocity to increase with increasing depth (which has never been reported under constant load, and would certainly not be expected). Of course, under displacement control, it would be possible to impose this condition (almost certainly resulting in the applied load having to be increased during the test). However, this has virtually never been done and the methodology being investigated here is based on the constant load condition that is in widespread use.

Usage of this methodology, which originated over 20 years ago (Mayo et al. 1990; Raman and Berriche 1992; Bower et al. 1993), has been, and continues to be, very extensive (Fujiwara and Otsuka 2001; Liu et al. 2007; Takagi et al. 2008; Mahmudi et al. 2009, 2013, 2015; Marques et al. 2013; Shen et al. 2013; Geranmayeh and Mahmudi 2014; Chinh and Szommer 2014; Kaur and Kaur 2014; Chatterjee et al. 2014; Wang and Zeng 2015; Nautiyal et al. 2015; Ma et al. 2015). In fact, the number of papers in which it has been employed now runs into many dozens, if not hundreds, and its usage actually appears to be accelerating. Values of $n$ obtained in this way are frequently interpreted in terms of creep mechanisms, even when they are implausibly high (>20), and despite the publication of several critical appraisals (Goodall and Clyne 2006; Chen and Bull 2009; Dean et al. 2014) of the method, which have exposed serious deficiencies and concerns. There are relatively few cases (Liu et al. 2007; Takagi et al. 2008; Mahmudi et al. 2009, 2013; Marques et al. 2013; Geranmayeh and Mahmudi 2014) in which it is claimed that agreement has been obtained between indentation-derived values of $n$ and those from conventional creep testing, and even these often relate to comparisons with literature data, rather than to systematic creep testing of the same material.

A partial explanation for its continued usage in the face of many warning signs may lie in the scope for confusion concerning the shape of penetration–time curves, which often bear at least a superficial resemblance to plots of creep strain against time, obtained from uniaxial (macroscopic) creep tests. Both types of curve exhibit an initial transient, followed by some kind of steady-state (constant gradient) region. In the case of conventional uniaxial creep data, the steady state regime commonly has a genuinely constant gradient over an extended period, associated with a microstructurally stable situation in which there is a well-defined rate-determining process, often involving the repeated surmounting of obstacles by dislocations. During indentation, however, the stress, strain and strain rate fields are complex, and vary continuously with position and time. Inevitably, primary creep is taking place in at least some locations throughout the test, which is commonly of relatively short duration. The net effect on the indenter velocity is difficult to predict (at least without using numerical methods), but there is no clear reason why the penetration rate should stabilise, or, if it does, why the behaviour should under those circumstances be dominated by stage II creep. In fact, it is now recognised that, even if the indentation velocity does stabilise, neglecting the effect of primary creep is likely to lead to major inaccuracies (Goodall and Clyne 2006; Chen and Bull 2009; Dean et al. 2014) in calculated values of $n$. The underlying point is that any similarity in shape between conventional creep curves and those obtained during indentation is largely coincidental and a stable indenter velocity does not in fact indicate that stage II creep is controlling the behaviour.

A previous paper (Dean et al. 2014) explored in some detail the reliability of the assumptions incorporated in the methodology, including use of “equivalent” stress and strain rate, and neglect of primary creep. The clear conclusion was reached that the method is fundamentally flawed and that inferred stress exponent values can vary widely for any given material. The current paper is focused on the (occasionally-proposed) contention that, provided the indentation test is run for long enough, and the penetration rate has become fully stable, the derived value of $n$ may be reliable.

## Indentation creep analysis

### Experimental procedures

Indentation runs were carried out with a Micromaterials Nanoindenter. Polished samples of OFHC copper were used. Two indenter tips were employed—a 50 μm diameter sphere and a standard Berkovich. The applied load in both cases was 500 mN and the creep dwell period was 1 hour. Full experimental details are given elsewhere (Dean et al. 2014).

### The analytical methodology

The steady state creep strain rate during uniaxial creep is commonly expressed as

1 $$ \frac{\mathrm{d}\varepsilon}{\mathrm{d}t} = C\sigma^{n} $$

where $\varepsilon$ is the creep strain, $C$ is a constant (with an Arrhenius dependence on temperature), $\sigma$ is the applied stress, $n$ is the stress exponent and $t$ is the time. The corresponding equation commonly applied to indentation creep is

2 $$ \frac{1}{h}\frac{\mathrm{d}h}{\mathrm{d}t} = C \biggl( \frac{P}{A_{\mathrm{p}} ( h )} \biggr)^{n} $$

where $h$ is the instantaneous indentation depth, $P$ is the applied load and $A_{\mathrm{p}}(h)$ is the projected contact area (as a function of $h$). The left hand term is the “equivalent” strain rate, while the bracketed term on the right hand side is the “equivalent” stress. The stress exponent, $n$, can thus be obtained as the gradient of a plot of the logarithm of the “equivalent” indentation strain rate against the logarithm of the “equivalent” indentation stress. It should be noted here that the value of $h$ is the total penetration depth, which will usually comprise the depth at the start of the creep dwell period, $h_{0}$, plus the cumulative penetration during that period, $h_{\mathrm{cr}}$.

Experimental penetration–time data can be handled in several different ways to obtain the indenter velocity. One is to use successive $h_{\mathrm{cr}}(t)$ data pairs, although this often results in excessive noise, making evaluation of the gradient a little difficult, and in practice this is rarely done. More common is to fit smooth (analytical) curves to these data. Power law relations are often used. This fitting procedure can be applied to the complete $h_{\mathrm{cr}}(t)$ data set, or just to the latter regions of the curve (where the penetration rate is stabilising). Such expressions can then be differentiated analytically (for any selected time).

## Illustrative application of the methodology

### Power law curve fitting

**Fig. 1:**
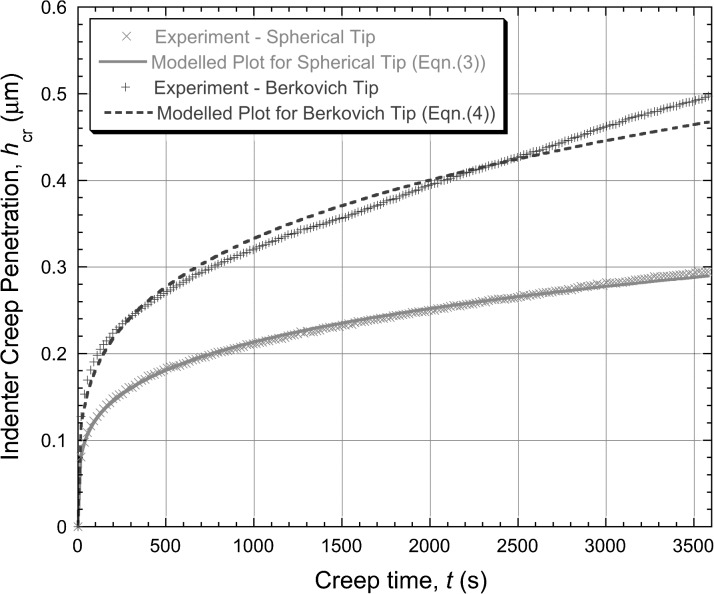
Experimental (creep) penetration—time plots for (creep) indentation into copper samples under a constant applied load of 500 mN, obtained with spherical and Berkovich indenters. Also shown are the best fit (power law) curves corresponding to Eqs. (3) and (4)

**Fig. 2:**
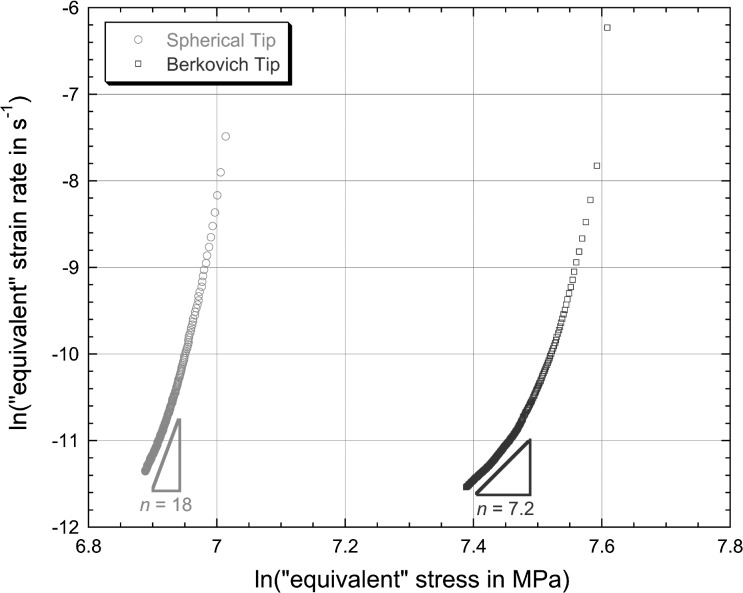
Plots, for the two sets of experimental data shown in Fig. 1, of the logarithm of the “equivalent” strain rate against the logarithm of the “equivalent” stress, obtained by applying Eq. (2) at a series of points along the modelled curves (Eqs. (3) and (4))

In Fig. 1, the experimental $h_{\mathrm{cr}}(t)$ data are presented and have also been fitted to simple power law functions, leading to the following best-fit expressions

3 $$\begin{aligned} &h_{\mathrm{cr}} = 0.04t^{0.24}\quad \mbox{and} \end{aligned}$$

4 $$\begin{aligned} &h_{\mathrm{cr}} = 0.053t^{0.27} \end{aligned}$$

for spherical and Berkovich tips, respectively. (In these equations, the values of the constants are those appropriate when $h_{\mathrm{cr}}$ is in μm and $t$ is in s.) It can be seen that the fits are good, particularly for the spherical tip. Corresponding plots of the logarithm of the “equivalent” strain rate against the logarithm of the “equivalent” stress, after application of Eq. (2) to the fitted curves, are shown in Fig. 2. It should be noted here that the total penetration, $h$, has been used in these equations, since it is this (rather than the creep penetration, $h_{\mathrm{cr}}$) that dictates the equivalent strain rates and stresses. The values of $h_{0}$ (to which $h_{\mathrm{cr}}$ is added to give $h$) were respectively 1.356 and 3.055 μm, for spherical and Berkovich indenters. As is commonly done, values of $n$ have been calculated from the gradient of these curves towards the latter stages of the test (as shown). Derived values of $n$ are 18 for the spherical indenter and 7.2 for the Berkovich indenter, as shown in the plot.

These are high values (representing implausibly high sensitivities of the strain rate to the stress) and they are, of course, significantly different for the two tip shapes. It is a little difficult to say whether these two curves do, in fact, appear to be stabilising (tending to a constant value of $n$), although they are certainly representative of many previous studies in which this assumption is made. Of course, the duration of the test (1 hour) is short compared with “conventional” creep testing, although, again, this is typical of many such indentation creep tests.

In fact, for the material concerned, conventional creep testing indicated that the “correct” value of $n$ during stage II creep was about 3.5, so both of the above values represent substantial over-estimates. The reasons for this were explored in some depth in the previous publication (Dean et al. 2014).

### Linear curve fitting

**Fig. 3:**
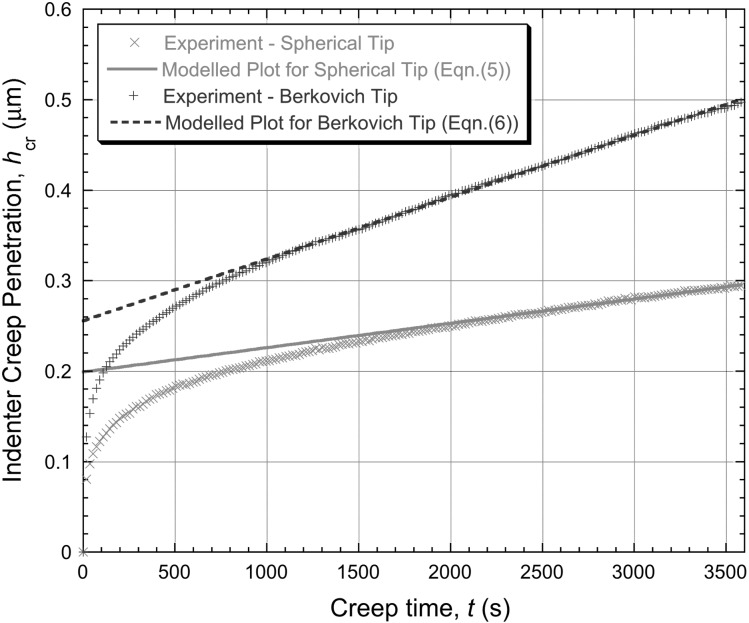
Experimental (creep) penetration—time plots for indentation into copper samples under a constant applied load of 500 mN, obtained with spherical and Berkovich indenters. Also shown are the best fit (linear) plots corresponding to Eqs. (5) and (6), which are designed to fit the gradient towards the end of the test

**Fig. 4:**
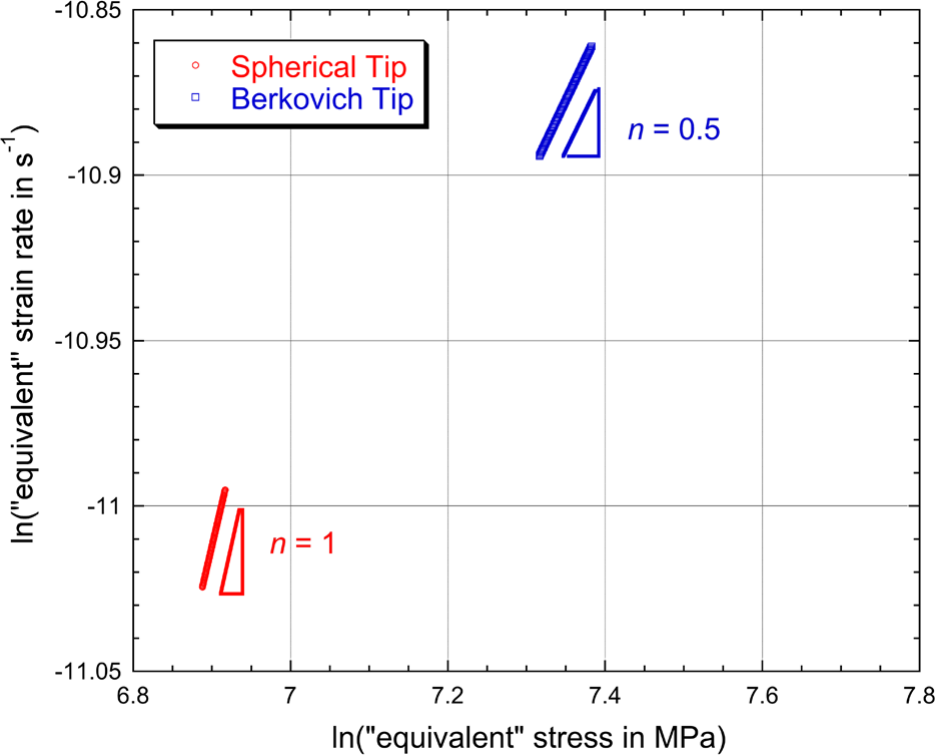
Plots, for the two sets of experimental data shown in Fig. 3, of the logarithm of the “equivalent” strain rate against the logarithm of the “equivalent” stress, obtained by applying Eq. (2) at a series of points (towards the end of the test) along the linear representation (Eqs. (5) and (6)).

While the procedure of Sect. 3.1 is commonly employed, it is in some ways more logical (and simpler) to just fit a linear plot to the penetration–time curve towards the end of the test. This has been done in Fig. 3, where, for the same raw experimental data, the latter stages have been fitted using linear functions, leading to the following best-fit expressions

5 $$\begin{aligned} &h_{\mathrm{cr}} = 0.199 + 0.000027t\quad \mbox{and} \end{aligned}$$

6 $$\begin{aligned} &h_{\mathrm{cr}} = 0.26 + 0.000068t \end{aligned}$$

for spherical and Berkovich tips, respectively, with the constants again relating to the case of $h_{\mathrm{cr}}$ being in μm and $t$ in s. These linear plots conform well in both cases to the measured data (towards the end of the test), reflecting the fact that the indenter velocity appears to have stabilised quite well. The corresponding plots of the logarithm of the “equivalent” strain rate against the logarithm of the “equivalent” stress, after application of Eq. (2) to the fitted curves (for $t >1800\ \mbox{s}$), are shown in Fig. 4. It can be seen that the values of $n$ obtained in this way appear to be about 1.0 and 0.5 for spherical and Berkovich tips, respectively. These values are clearly inconsistent with those obtained in Fig. 2, despite the good agreement between experimental data and modelled curves in Fig. 1.

## Evaluation of n for a constant indenter velocity

### General case

While the outcome of Fig. 4 looks surprising at first sight, it is in fact quite straightforward to demonstrate that it follows inevitably from the assumption of a truly stable indenter velocity (irrespective of the “correct” value of $n$—in fact, irrespective of any characteristics of the actual creep behaviour!).

Equation (2) can be expressed in logarithmic form as follows:

7 $$ \ln \biggl( \frac{1}{h}\frac{\mathrm{d}h}{\mathrm{d}t} \biggr) = \ln C + n\ln \biggl( \frac{P}{A_{\mathrm{p}}} \biggr). $$

The stress exponent is thus given by

8 $$ n = \frac{\partial [ \ln ( \frac{1}{h}\frac{\mathrm{d}h}{\mathrm{d}t} ) ]}{\partial [ \ln ( \frac{P}{A_{\mathrm{p}}} ) ]}. $$

### Spherical indenter tip

The necessary manipulation of Eq. (8) is now quite simple. The value of $P$ is constant throughout, so the only change in the denominator during a test comes from the dependence of the projected area on the (total) penetration depth, $h$. For a sphere, this relationship is

$$A_{\mathrm{p}} = \pi \bigl( 2Rh - h^{2} \bigr) $$

so that Eq. (8) can be written

9 $$\begin{aligned} &n = \frac{\partial [ \ln ( \frac{\mathrm{d}h}{\mathrm{d}t} ) - \ln ( h ) ]}{\partial [ \ln ( \frac{P}{\pi} ) - \ln ( 2Rh - h^{2} ) ]}, \end{aligned}$$

10 $$\begin{aligned} &\therefore n = \frac{\partial [ \ln ( \frac{\mathrm{d}h}{\mathrm{d}t} ) - \ln ( h ) ]}{\partial [ \ln ( \frac{P}{\pi} ) - \ln ( h ) - \ln ( 2R - h ) ]}. \end{aligned}$$

The value of $P/\pi$ is constant. Provided that (as in the present case) $R\gg h$, the value of ($2R - h$) is also approximately constant. It follows that, for a situation in which the indenter velocity ($\mathrm{d}h/\mathrm{d}t$) has become constant, this equation reduces to

11 $$ \therefore n \approx \frac{\partial [ - \ln ( h ) ]}{\partial [ - \ln ( h ) ]} = 1. $$

This is consistent with the plot in Fig. 4. In fact, all values of $n$ derived from linear fits to experimental penetration–time data for spherical indenter tips will be ∼1. (Even if the tip diameter is comparable in magnitude to the penetration depth, which would be unusual, the value of $n$ will still be quite close to unity.)

### Berkovich indenter tip

This case is similar to the above, except that the projected area is now given by

$$A_{\mathrm{p}} = 24.5h^{2} $$

so that Eq. (8) can in this case be written

12 $$ n = \frac{\partial [ \ln ( \frac{\mathrm{d}h}{\mathrm{d}t} ) - \ln ( h ) ]}{\partial [ \ln ( \frac{P}{24.5} ) - 2\ln ( h ) ]}. $$

The analysis is thus very similar to that in Sect. 4.2, leading to

13 $$ \therefore n \approx \frac{\partial [ - \ln ( h ) ]}{\partial [ - 2\ln ( h ) ]} = 0.5. $$

For this type of (self-similar) indenter tip, the projected area is always proportional to the square of the penetration depth, so the inferred value of $n$, for a truly stable indenter velocity, will always be 0.5. This is also consistent with the plot in Fig. 4.

### Area-based analysis

**Fig. 5:**
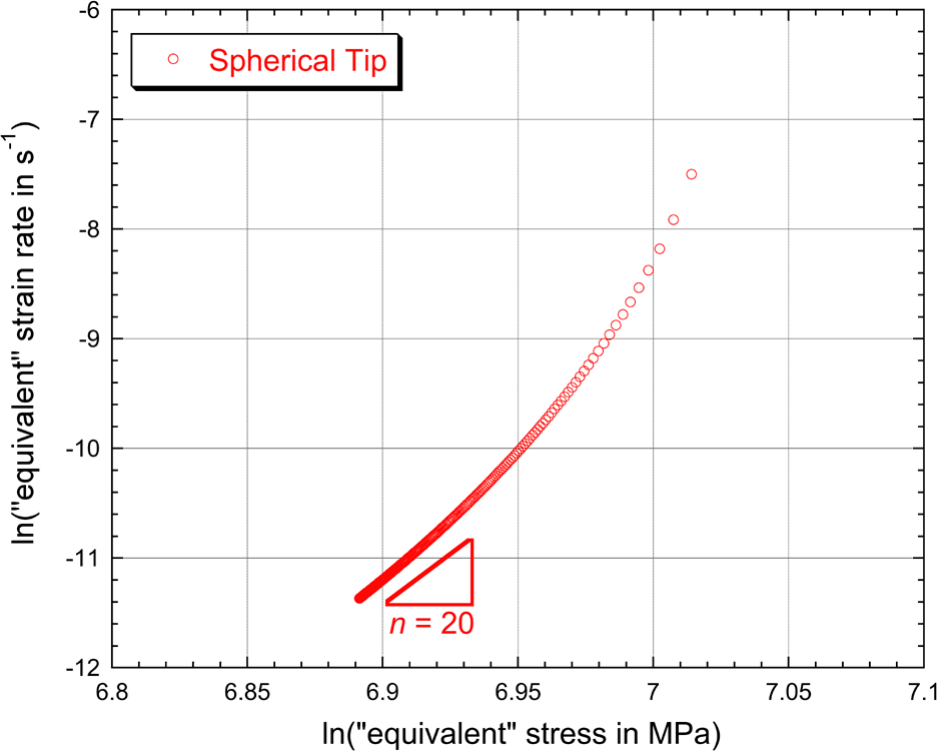
Plot, for the experimental data in Fig. 1 corresponding to the spherical tip, of the logarithm of the “equivalent” strain rate (area-based version) against the logarithm of the “equivalent” stress, obtained by applying Eq. (14) at a series of points along the modelled curves (Eqs. (3) and (4))

**Fig. 6:**
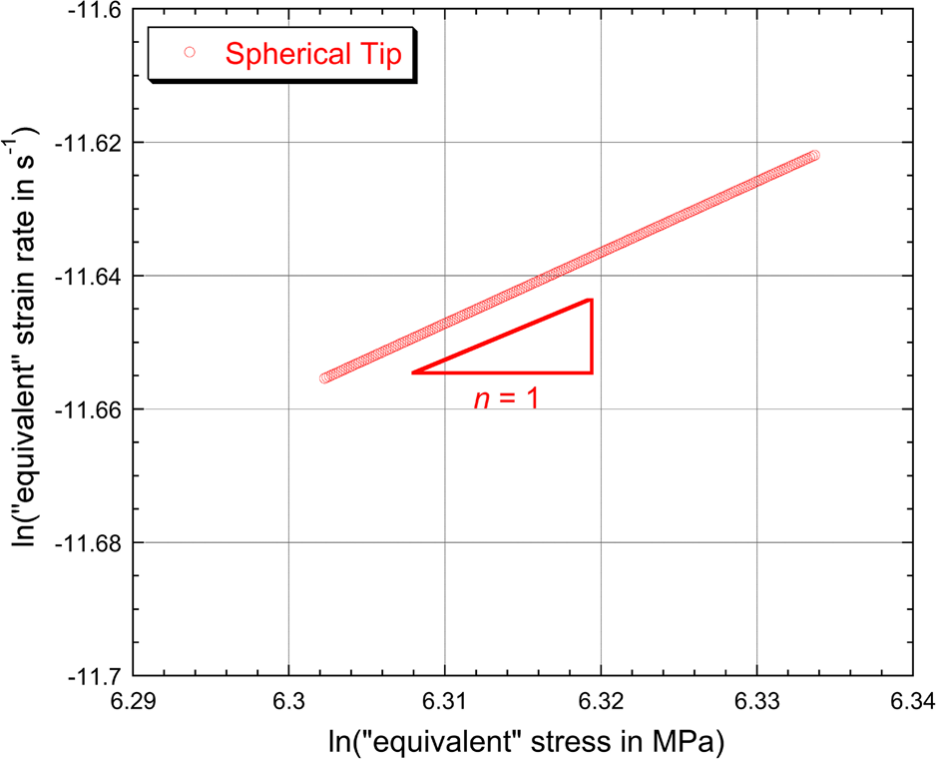
Plot, for the experimental data in Fig. 1 corresponding to the spherical tip, of the logarithm of the “equivalent” strain rate (area-based version) against the logarithm of the “equivalent” stress, obtained by applying Eq. (14) at a series of points (towards the end of the test) along the linear representation (Eqs. (5) and (6))

Lucas and Oliver (1999) suggested that a more appropriate definition of the indentation strain rate, for a non-self-similar indenter tip (such as a sphere), is the rate of change of (projected) contact area divided by the instantaneous (projected) contact area—that is to say, $((\mathrm{d}A_{\mathrm{p}}/\mathrm{d}t)/A_{\mathrm{p}})$, rather than $((\mathrm{d}h/\mathrm{d}t)/h)$. (These two expressions are equivalent for a self-similar tip shape.) The rationale supplied for this is that it is the rate at which the elastic/plastic boundary moves that is determined by the creep behaviour of the sample. The equivalent of Eq. (2) in this case is thus

14 $$ \biggl( \frac{1}{A_{\mathrm{p}}} \biggr) \biggl( \frac{\mathrm{d}A_{\mathrm{p}}}{\mathrm{d}t} \biggr) = C \biggl( \frac{P}{A_{\mathrm{p}}} \biggr)^{n} $$

where $A_{\mathrm{p}}$ is, of course, a function of $h$. The plots of Figs. 2 and 4 have been recreated (for the spherical tip only), using this relationship in place of Eq. (2). The corresponding outcomes are shown in Figs. 5 and 6. It can be seen that, for this definition of the indentation strain rate, using the power law expression leads to a different (but still unreasonable) value for $n$, while the linear fit leads to $n = 1$, as before. Of course, for a self-similar tip shape, using this definition of the indentation strain rate has no effect on the analysis. It is therefore clear that the exact definition of the equivalent strain rate has no significant effect on the outcome.

### Effect of allowing penetration depth to approach tip radius

The analysis of Sect. 4.2, for the spherical tip, involved the assumption that $R\gg h$. Typical radii for spherical tips are at least about 5–10 μm, and they rarely penetrate more than a few microns, so this condition commonly applies. However, in the interest of completeness, it is worth noting whether the analysis should be changed in any way if this condition is relaxed and the tip is allowed to penetrate up to $h = R$ (i.e. to a depth where the sample surface has reached the “equator” of the sphere). This also allows an estimate of the range of $h/R$ values over which the “$R\gg h$” condition effectively applies.

**Fig. 7:**
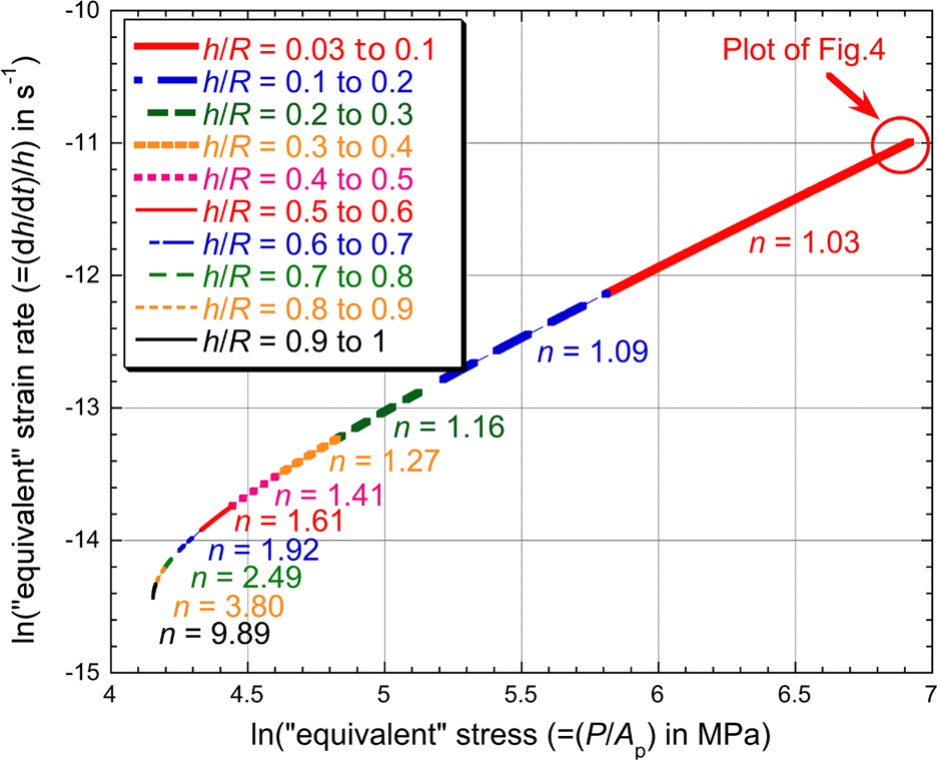
Plot, based on the linear representation of the experimental data in Fig. 1 for the spherical tip (Eq. (5)), of the logarithm of the “equivalent” strain rate against the logarithm of the “equivalent” stress, obtained by applying Eq. (14) at a series of points. This plot is taken well beyond the limit of the actual experimental data (Fig. 4), to the regime where the penetration depth ($h$) approaches the tip radius ($R$)

The outcome of this investigation is shown in Fig. 7. This is an equivalent plot to that of Fig. 4 (spherical indenter case), using the same (linear) fit (Eq. (5)), but extending the plot from the actual limit of the experimental data up to $h = 50\ \upmu \mbox{m}$ $(h/R = 1)$. The assumption is thus made that the penetration velocity remains constant (at 0.027 nm s^−1^—see Eq. (5)) for the further $1.8 \times 10^{6}\ \mbox{s}$ (∼21 days) needed to reach this depth. As expected, the gradient of this plot is close to unity ($n \approx 1$) over the portion corresponding to the early stages (where both the equivalent strain rate and the equivalent stress have higher values). It is also clear that this regime extends up to relatively high values of $h/R ({\sim} 0.3)$—covering virtually all plausible experimental cases. It is true that, as $h/R$ exceeds this value, this “limiting” gradient (measured $n$ value) starts to increase and, as $h$ becomes close to $R$, it becomes relatively large (∼10). The reasons for this are clear when the effect of the geometry is considered. Of course, these “limiting” values still bear no relation whatsoever to the creep characteristics of the material—they are simply a function of the conditions chosen for the test. In any event, these increases in the limiting value of $n$ only occur under conditions outside the range of “conventional” testing, which “should” yield $n = 1$. For a self-similar tip shape, $n = 0.5$ is expected for any penetration depth.

### Interpretation of outcome

Study of the literature does reveal a number of cases (Nautiyal et al. 2015; Huang et al. 2009; Choi et al. 2011; Wu et al. 2013; Machaka et al. 2014), covering a wide range of materials, in which inferred values of $n$ have been in the range “normally” expected in view of the above analysis (∼1 with spherical tips and ∼0.5 for Berkovich, etc.), although, it is perhaps surprising that there are not more examples. Of course, this outcome is entirely independent of the actual creep mechanism, so it is clearly of concern in this context that such values have commonly been taken to be indicative of diffusional (Nabarro–Herring or Coble) creep. In fact, since indentation always tends to create conditions (at least in the vicinity of the tip) in which the deviatoric (von Mises) stress is relatively high (close to the yield stress), there is a strong tendency for indentation creep to occur via dislocation motion (usually leading to a value of $n$ in the range 2–5) and diffusional creep is inherently unlikely. Of course, the above inferences (based on a value of $n$ that should be obtained whenever the indenter velocity has become truly stable) would clearly be incorrect in any event.

It can thus be concluded that there is a stark choice when applying these analytical procedures to indentation creep data. Either the (most common) route (of representing the data by a functional expression) can be followed, in which case the outcome is highly unpredictable and variable, but is likely to be a substantial overestimate, or the supposition of a constant indenter velocity can be rigorously imposed (and measured towards the end of the test), in which case a value of either 1.0 or 0.5 will be obtained, depending on tip shape (but independent of the measured velocity value, or indeed of any creep characteristics of the material). What the methodology certainly cannot deliver is an estimate of $n$ that bears any systematic relation to the true value of the stress exponent for steady state creep. It is now completely clear that its usage should be discontinued.

An obvious question to ask under these circumstances is how such extensive usage of the methodology can possibly have persisted over such a long period. Part of the answer certainly lies in the development of data processing tools that have made the procedure simple, quick and convenient, while apparently also being widely accepted and established. Another relevant factor concerns the nature of certain sensitivities. Clearly, whatever formulation is used to represent the experimental data, its gradient should become constant at long times—this is the basis of the whole concept. However, in practice this commonly turns out to require times that are considerably longer than the duration of the test. When plotted using these logarithmic functions, it often appears that some sort of stabilisation has occurred in the value of $n$, when in fact it has not. As the analysis presented here has demonstrated, actually imposing a constant gradient reveals that all such manipulations of the experimental data are in fact tending to a universal value of $n$ that bears no relation to the creep behaviour of the material. Evidently, there are salutary lessons to be learned from the extended time that it has taken for this to become clear.

To finish on a more positive note, the (many hundreds of) existing indentation creep data sets probably do, at least in some cases, incorporate information that could be interpreted to obtain meaningful creep characterisation parameters (provided that relevant data concerning tip size and shape, load histories etc have been retained). All that is required is implementation of suitable inverse FEM modelling procedures, so as to converge iteratively on best fit values. These parameters will certainly include those describing primary creep, as well as secondary creep. However, even in (probably common) cases where primary creep is completely dominating the indentation response, relationships between creep rates in the two regimes may allow evaluation of steady state parameters. As such procedures become better established and easier to implement, identification of optimal comparator data sets will become clearer. These may involve usage of multiple applied loads and tip shapes, but it’s certainly possible that some existing data sets would be adequate for this purpose without further experimental work being necessary.

## Conclusions

The following conclusions can be drawn from this work, which relates to indentation creep:

- Illustrative data have been presented from instrumented indentation of pure copper samples at room temperature, obtained using both spherical and Berkovich tip shapes, and an established methodology has been employed, to infer from these data the value of the (steady state) stress exponent, $n$. This led to values of about 18 and 7, respectively, for these two tip shapes (under the conditions employed). The value obtained by conventional creep testing of this material was about 3.5. It is a common observation that indentation-derived values of $n$ obtained in this way are often variable and are usually over-estimates.
- The methodology is commonly implemented by fitting an analytical curve to the penetration–time data, but it is based on the concept of the indenter penetration rate reaching a constant value (“steady state” condition) at relatively long times. By actually taking the velocity as constant, and evaluating it towards the end of the tests, values of $n$ were obtained as 1.0 and 0.5, respectively, for spherical and Berkovich tips. This is the case for both depth-based and area-based expressions for the “indentation strain rate”.
- Using a simple mathematical analysis, it is shown that these values (1.0 and 0.5) will always be obtained if the indenter velocity is taken to have reached a genuinely constant value (and will exhibit no dependence on the actual measured velocity). It is thus evident that the methodology is fundamentally flawed and it is now completely clear that any similarity between values of $n$ obtained using these simple analytical manipulations of indentation creep data and the true steady state stress exponent of the material must be entirely fortuitous.
